# Evaluating the Prevalence and Distribution of Dental Anomalies in the Permanent Dentition of Patients Seeking Dental Care

**DOI:** 10.7759/cureus.30156

**Published:** 2022-10-10

**Authors:** Manish S Dagdiya, Amesh Golwara, Niharika Shahi, Deep Sundar, Abhishek Sinha, Lalima Kumari

**Affiliations:** 1 Department of Orthodontics and Dentofacial Orthopaedics, Saraswati-Dhanwantari Dental College and Hospital, Parbhani, IND; 2 Department of Orthodontics, Buddha Institute of Dental Sciences and Hospital, Patna, IND; 3 Department of Pedodontics and Preventive Dentistry, Purvanchal Institute of Dental Sciences, Gorakhpur, IND; 4 Department of Public Health Dentistry, Buddha Institute of Dental Sciences and Hospital, Patna, IND; 5 Department of Dentistry, Patna Medical College and Hospital, Patna, IND; 6 Department of Orthodontics, Patna Dental College and Hospital, Patna, IND

**Keywords:** prevalence, peg-shaped, microdontia, hypodontia, dental anomalies

## Abstract

Background: Disturbances seen during tooth formation result in developmental dental anomalies presenting in the oral cavity. These anomalies manifest as discrepancies in the number, color, size, and shape of the teeth. These dental anomalies can either be acquired, congenital, or developmental. Their early detection and management are necessary as they affect aesthetics and occlusion. The study had the aim of gauging the prevalence of developmental anomalies in the permanent dentition of Indian subjects.

Methods: A total of 1192 participants recruited from the institute for study purposes, comprising males and females, were examined clinically and radiographically, and their dental casts were also evaluated. These subjects were assessed for anomalies in position, structure, number, and/or shape. Anomalies in the position include transmigration, transportation, and/or ectopic position; anomalies in the structure, including dentinogenesis imperfecta or amelogenesis imperfecta; anomalies in number, including hyperdontia or hypodontia; and anomalies in shape, including peg laterals, taurodontism, fusion, dens evaginatus, talon cusp, and/or microdontia.

Results: A statistically significant difference was seen in unilateral microdontia and dentinogenesis imperfecta between males and females, with attained p-values of 0.003 and 0.06, respectively. The results of the present study showed that 9.89% (n = 118) study subjects, whereas 1% (n = 12) study subjects had two dental anomalies in their permanent dentitions, with no subject presenting more than two dental anomalies, showing that various dental anomalies have a low prevalence in the Indian population.

Conclusion: The present study has led to the conclusion that the prevalence of dental anomalies is low in Indian subjects. However, these anomalies should be detected and treated early to prevent them from causing further complications.

## Introduction

Disturbances during tooth formation result in developmental dental anomalies presenting in the oral cavity. These anomalies manifest as discrepancies in the number, color, size, and shape of the teeth. These dental anomalies can be acquired, congenital, or developmental [1]. These anomalies can be seen in single teeth, teeth groups or a few teeth, or all teeth/complete dentition. These anomalies can be associated with some systemic diseases. These dental anomalies have a very low prevalence compared to periodontal diseases and dental caries [2]. However, their early detection and management are necessary as they affect aesthetics and occlusion. Previous literature data suggest that growth and mineralization lead to differences in the developed teeth.

As described in the literature [3], developing teeth clearly differ in how they mineralize and grow. Various epidemiologic studies are being carried out in the literature in different geographic areas of the globe to gauge the types and prevalence of dental anomalies in different populations. However, such studies focusing on the Indian population are scarce in the literature [4].

These developmental anomalies in teeth can be seen due to either environmental or genetic factors or a combination of both genetic and environmental factors [5]. These dental anomalies can be seen as an evolutionary trend. These developmental dental tooth anomalies can occur during morphological differentiation of the teeth, leading to anomalies in the position, shape, and number of the teeth. Changes in dental normalcy can also be caused by systemic diseases [6].

The incidence and prevalence of these developmental tooth anomalies can show insight into vital information for genetic studies and can help in understanding the differences between various populations [7]. It is important to find and treat these developmental tooth abnormalities as soon as possible because they can affect the way your teeth look, make you more likely to get cavities, cause problems with your gums, make it hard to chew or cause malocclusion [7]. In the past researchers looked at how common developmental problems with teeth were in different sample groups and populations. However, this research has reported conflicting results. These differences in the data can be explained by the different sampling methods, investigations, geographic areas looked at, and criteria and parameters for the investigations [6-8].

One literature study was done in 2014, where authors searched the previous literature and concluded that the prevalence of supernumerary teeth shows a variation of 0.04% to 2.29%. Another study conducted in 2022 reported that dental hard tissue anomalies have large variations in different countries and setups [9]. In addition, the literature data also showed that more prevalence of hypodontia is seen in deciduous teeth compared to permanent teeth, with a frequency range of 0.03% to 10.1% as depicted by different studies. Very little research has been done on how common other dental abnormalities are, like laceration, which has a prevalence range of about 6% to 16% [10].

The studies conducted previously detailed these anomalies as having predilections for a few teeth compared to others [6-8]. A few studies have found that third molars, second premolars (both maxillary and mandibular), maxillary lateral incisors, and mandibular central incisors are the teeth that people are most likely to be missing [9-11]. Many studies have reported that the most commonly seen supernumerary teeth are mesiodens, whereas another group of studies shows that the prevalence of dental anomalies is governed by gender [9-11]. A few other studies disregard this fact. Also, a few other studies showed that the effect of gender is based on the type of anomaly being assessed [10-13].

Due to the lack of information about how dental abnormalities develop in the Indian population, this survey study was done at a Dental Care Institute to find out what kinds of dental abnormalities are common in the Indian population and how common they are. The present study had the aim of gauging the prevalence of developmental anomalies in the permanent dentition of Indian subjects.

## Materials and methods

This longitudinal epidemiological survey study was commenced to gauge the prevalence of developmental anomalies in the dentition of Indian subjects. The study was carried out at Saraswati-Dhanwantari Dental College and Hospital College, Parbhani, Maharashtra after ethical committee clearance was taken from the Institute (SDDC/IERBC/0238). The participants were patients from the outpatient departments of the institute. The study participants were 1192 subjects, including both males and females. Informed consent was taken from all the participants in both verbal and written formats. The inclusion criteria for the study were subjects willing to participate, had at least one dental anomaly, and were in a sound mental state to consent. The exclusion criteria were subjects managed by prosthetic rehabilitation for the anomalies, those who underwent any treatment of the affected teeth theta might alter size and shape, history of trauma to the affected area, and subjects who did not consent to the study participation. The study was carried out for a period of one year.

For all the participants, the complete intraoral examination was done by a single examiner with expertise in the field. Prior to the intraoral examination, detailed demographic data and medical history were collected. The intraoral examination was done under adequate light using a mouth mirror and an explorer. The whole dentition for all subjects was critically evaluated for anomalies in shape, size, or number of teeth. This was followed by radiographic evaluation wherever feasible. The radiographs taken were orthopantomograms or IOPARs (intraoral periapical radiographs).

On intraoral examination, the following dental anomalies were found: anomalies in position including transmigration, transportation, and/or ectopic position; anomalies in the structure including dentinogenesis imperfecta or amelogenesis imperfecta; anomalies in number including hyperdontia or hypodontia; and anomalies in shape including peg laterals, taurodontism, fusion, dens evaginatus, talon cusp, and/or microdontia. For anomaly reporting, the criteria by Gupta et al. in 2011 [14] were followed which is standardized.

Statistical assessment of extracted data was done using SPSS software version 21.0 (IBM Corp., Armonk, NY) and a t-test along with one way ANOVA test. The data were presented as a mean and standard deviation, as well as a number and a percentage. The p-level of 0.05 was taken as a cut-off for significance.

## Results

The present study showed that 9.89% (n=118) study subjects, whereas 1% (n=12) study subjects had two dental anomalies in their dentition, with no subject presenting more than two dental anomalies, showing various dental anomalies have a low prevalence in the Indian population.

On gender-based analysis, the study results showed that unilateral microdontia was significantly lesser in males (n=4) compared to females (n=49). This was a statistically remarkable difference with p=0.003. Bilateral microdontia had an equal distribution in both genders and was reported in six males and six females, which was statistically non-significant with p=1.000. Similar equal distribution was seen for macrodontia where unilateral microdontia was seen in one male and one female each, which also showed statistically non-significant results with p=1.000. The distribution of bilateral macrodontia among males and females also showed statistically non-significant results with p=0.14. No case of transmigration or transportation was seen in any subject of either gender (Table 1).

**Table 1:** Dental anomalies in study subjects related to tooth shape and structure

S. No	Anomalies	Females	Males	Total	p-value
1	Unilateral microdontia	4	49	53	0.003
2	Bilateral microdontia	6	6	12	1.000
3	Unilateral macrodontia	1	1	1	1.000
4	Bilateral macrodontia	2	3	5	0.14
5	Unilateral Transmigration	0	0	0	-
6	Bilateral Transmigration	0	0	0	-
7	Unilateral Transportation	0	0	0	-
8	Bilateral Transportation	0	0	0	-

Concerning anomalies related to tooth number, hypodontia was seen in six study subjects where no hypodontia was seen in the mandibular central incisor, maxillary molar, and maxillary central incisor. Hypodontia in the maxillary lateral incisor was observed in one female and no male, resulting in statistically insignificant findings with p=0.13. In maxillary premolars, hypodontia was seen in two males and one female, which also showed statistically non-remarkable results with p=0.42. In the mandibular arch, one male and one female had hypodontia of the premolar, whereas only one female had hypodontia of the mandibular molar. These differences were non-significant on statistical grounds with p=0.14. In one female and two males in the present study, bilateral maxillary hyperdontia was seen in one male and one female in the present study. These contrasts were non-significant on statistical considerations, with p-values of 0.15 and 0.34 respectively. Mandibular hyperdontia unilaterally was seen in one female and two males with statistically non-significant results (p=0.13). In the present study, one male and one female were found to have bilateral mandibular hyperdontia. This was non-significant statistically (p=0.15) as depicted in Table 2.

**Table 2:** Dental anomalies in study subjects related to tooth number

S. No	Anomalies	Females	Males	Total	p-value
1	Hypodontia				
a	Maxillary central incisor	0	0	0	-
b	Maxillary lateral incisor	1	0	1	0.13
c	Maxillary premolar	0	1	2	0.42
d	Maxillary molar	0	0	0	-
2	Hypodontia				
a	Mandibular central incisor	0	0	0	-
b	Mandibular premolar	1	1	2	0.14
c	Mandibular molar	1	0	1	0.14
3	Hyperdontia maxillary unilateral	1	2	3	0.15
4	Hyperdontia maxillary bilateral	1	1	2	0.34
5	Hyperdontia mandibular unilateral	1	2	3	0.13
6	Hyperdontia mandibular bilateral	1	1	2	0.15

On evaluating the dental anomalies related to tooth shape and structure, amelogenesis imperfecta was found in one female and four males with a statistically non-significant difference (p=0.24). Dentinogenesis imperfecta was observed in one female and three males. This was statistically significant with p=0.06. Unilateral and bilateral talon cusps were seen in two and three study subjects respectively, which was statistically non-significant between the two genders, with respective p-values of 0.34 and 0.13. Unilateral and bilateral dens evaginatus were noticed in two and three study subjects, respectively. This varies by gender and was statistically significant for unilateral (p=0.08) and bilateral dens evaginatus (p=0.45). Fusion unilaterally was seen in one male and one female, and bilateral fusion in one male subject. These differences were non-remarkable statistically, with the respective p-values of 0.33 and 0.14, respectively. In one female and two males in the present study, a statistically non-significant difference with p=0.43 was found. In this study, bilateral peg lateral was seen in five females and seven males. This difference also produced non-significant results statistically with p=0.34 (Table 3).

**Table 3:** Dental anomalies in study subjects related to tooth shape and structure

S. No	Anomalies	Females	Males	Total	p-value
1	Amelogenesis Imperfecta	1	4	5	0.24
2	Dentinogenesis Imperfecta	1	3	4	0.06
3	Unilateral Talon cusp	1	1	2	0.34
4	Bilateral Talon cusp	1	2	3	0.13
5	Unilateral Dens evaginatus	1	1	2	0.08
6	Bilateral Dens evaginatus	1	2	3	0.45
7	Unilateral Fusion	1	1	2	0.33
8	Bilateral Fusion	1	0	1	0.14
9	Unilateral Peg lateral	1	2	3	0.43
10	Bilateral Peg lateral	5	7	12	0.34

## Discussion

The findings of the study indicated that there is a significant difference between the rates of dental abnormalities that are seen in males and females. Unilateral microdontia and dentinogenesis imperfecta showed a significant difference between males and females based on statistical grounds, with p-values of 0.003 and 0.06, respectively. The current study found that dental anomalies were present in 9.89% of the study subjects. However, only 1% of the study subjects had two dental anomalies in their dentition, and no subject presented more than two dental anomalies. This indicates that various dental anomalies have a low prevalence in the Indian population. These findings were comparable to those published by Ghabanchi et al. [13] in 2010 and by Gupta et al. [14] in 2011. The authors of those studies discovered, among other things, that the Indian population has a relatively low prevalence of various dental abnormalities.

The findings of the study also revealed that a condition known as unilateral microdontia was present in four males and 49 females. With a p-value of 0.45003, this difference was found to have statistically significant variations. It was determined that the presence of bilateral microdontia in six males and six females did not constitute a statistically significant difference (p=1.000). In the current study, bilateral maxillary hypodontia was observed in one male and one female participant, whereas unilateral maxillary hypodontia was observed in two male participants. The p-values for these differences were 0.15 and 0.34, respectively, indicating that they did not represent a statistically significant difference. One female and two males were observed to have unilateral mandibular hyperdontia, but these findings were not considered statistically significant (p=0.13). The current study found a case of bilateral mandibular hyperdontia in one male and one female participant. This failed to reach the threshold of statistical significance (p = 0.15). These findings were consistent with those found by Albashaireh and Khader [15] and Guttal et al. [16] in 2006 and 2010, respectively, where tooth number anomalies including hyperdontia and hypodontia had similar incidence and prevalence to those found in the present study. Additionally, dentinogenesis imperfecta was observed in one female and three males.

Two study subjects had unilateral talon cusps, and three subjects had bilateral talon cusps. This difference was not statistically significant between males and females, with p-values of 0.34 and 0.13 for males and females, respectively. These findings were comparable to the study by Prabhu et al. in 2012 where authors reported the prevalence of talon cusp to be 0.58% in their study done in India [17]. Two of the study subjects had unilateral dens evaginatus, while three had bilateral dens evaginatus. According to the statistical analysis, these differences based on gender were significant for unilateral (p=0.08) and bilateral dens evaginatus (p=0.45). These results were in agreement with the recent study of 2022 done by Jain et al. 2022 in their studies, the authors reported the prevalence of dens evaginatus to be 1.26% with 0.73% males and 0.53% females showing male predilection [18]. One of the female participants and two of the male participants in this study showed a statistically non-significant difference with a p-value of 0.43. In the current study, bilateral peg lateral was seen in five females and seven males. The condition was more common in males. With a p-value of 0.34, this difference was also not statistically significant. When Ezoddini et al. [19] and Malcic et al. [20] looked at the teeth of their study subjects in 2007 and 2006, respectively, they came to similar conclusions. These results were similar to what they found.

This study had several shortcomings, including the limited sample size, a cross-sectional design, and potential for bias caused by the study's geographical setting; as a result, additional long-term studies are required. The present study suggested the frequency of a subset of dental abnormalities; however, future research should investigate the frequency of all types of dental abnormalities.

## Conclusions

The present study, considering its shortcomings, concludes that the prevalence of dental anomalies is low in Indian subjects. However, these anomalies should be detected and treated early to prevent them from causing further complications. An early diagnosis of dental anomalies can prevent some aesthetic, orthodontic, and periodontal problems. Knowledge of the prevalence and distribution of the anomalies can help clinicians detect these anomalies at an earlier stage.
